# A novel evaluation benchmark for medical LLMs illuminating safety and effectiveness in clinical domains

**DOI:** 10.1038/s41746-025-02277-8

**Published:** 2025-12-26

**Authors:** Shirui Wang, Zhihui Tang, Huaxia Yang, Qiuhong Gong, Tiantian Gu, Hongyang Ma, Yongxin Wang, Wubin Sun, Zeliang Lian, Kehang Mao, Yinan Jiang, Zhicheng Huang, Lingyun Ma, Wenjie Shen, Yajie Ji, Yunhui Tan, Chunbo Wang, Yunlu Gao, Qianling Ye, Rui Lin, Mingyu Chen, Lijuan Niu, Zhihao Wang, Peng Yu, Mengran Lang, Yue Liu, Huimin Zhang, Haitao Shen, Long Chen, Qiguang Zhao, Si-Xuan Liu, Lina Zhou, Hua Gao, Dongqiang Ye, Lingmin Meng, Youtao Yu, Naixin Liang, Jianxiong Wu

**Affiliations:** 1 Medlinker Intelligent and Digital Technology Co. Ltd., Beijing, China; 2 Peking University School of Stomatology, Haidian, Beijing, China; 3 Department of Rheumatology and Clinical Immunology, Peking Union Medical College Hospital, Chinese Academy of Medical Sciences and Peking Union Medical College, Beijing, China; 4 Center of Endocrinology, National Center of Cardiology & Fuwai Hospital, Chinese Academy of Medical Sciences and Peking Union Medical College, Beijing, China; 5 Department of Psychological Medicine, Peking Union Medical College Hospital, Chinese Academy of Medical Sciences and Peking Union Medical College, Beijing, China; 6 Department of Thoracic Surgery, Peking Union Medical College Hospital, Chinese Academy of Medical Sciences and Peking Union Medical College, Beijing, China; 7 Department of Respiratory and Critical Care Medicine, the 8th Medical Center of PLA General Hospital, Beijing, China; 8 Department of Obstetrics & Gynecology, the Fourth Medical Center of PLA General Hospital, Beijing, China; 9 Shuguang Hospital Affiliated to Shanghai University of Traditional Chinese Medicine, Shanghai, China; 10 Department of Urology, The Second Affiliated Hospital of Harbin Medical University, Harbin, Heilongjiang Province China; 11 Department of Radiation Oncology, Harbin Medical University Cancer Hospital, Harbin, Heilongjiang Province China; 12 Department of Dermatology, Shanghai Skin Disease Hospital, Tongji University School of Medicine, Shanghai, China; 13 Department of Oncology, East Hospital Affiliated to Tongji University, Tongji University School of Medicine, Tongji University, Shanghai, China; 14 General Surgery Department, Tongji Hospital, School of Medicine, Tongji University, Shanghai, China; 15 Department of Neurosurgery, Huashan Hospital, Shanghai Medical College, Fudan University, Shanghai, China; 16 Department of Ultrasound, National Cancer Center/National Clinical Research Center for Cancer/Cancer Hospital, Chinese Academy of Medical Sciences and Peking Union Medical College, Beijing, China; 17 Department of Hepatobiliary Surgery, National Cancer Center/National Clinical Research Center for Cancer/Cancer Hospital, Chinese Academy of Medical Sciences and Peking Union Medical College, Beijing, China; 18 Department of General Surgery, The Fourth Affiliated Hospital of Xinjiang Medical University, Urumqi, Xinjiang Uygur Autonomous Region China; 19 Department of Otolaryngology-Head and Neck Surgery, Shanxi Bethune Hospital, Shanxi Academy of Medical Sciences, Tongji Shanxi Hospital, Third Hospital of Shanxi Medical University, Taiyuan, Shanxi Province China; 20 Department of Clinical Laboratory, Seventh People’s Hospital of Shanghai University of Traditional Chinese Medicine, Shanghai, China; 21 Department of Orthopedics, Guangzhou Red Cross Hospital of Jinan University, Guangzhou, Guangdong Province China; 22 Department of Imageology, Anzhen Hospital. Capital Medical University, Beijing, China; 23 Beijing EuroEyes, Beijing, China; 24 Department of Interventional Radiology, the Fourth Medical Center of Chinese PLA General Hospital, Beijing, China

**Keywords:** Computational biology and bioinformatics, Diseases, Health care, Medical research

## Abstract

Large language models (LLMs) hold promise in clinical decision support but face major challenges in safety evaluation and effectiveness validation. We developed the Clinical Safety-Effectiveness Dual-Track Benchmark (CSEDB), a multidimensional framework built on clinical expert consensus, encompassing 30 metrics covering critical areas like critical illness recognition, guideline adherence, and medication safety, with weighted consequence measures. Thirty-two specialist physicians developed and revised 2069 open-ended Q&A items aligned with these criteria, spanning 26 clinical departments to simulate real-world scenarios. Benchmark testing of six LLMs revealed moderate overall performance (average total score 57.2%, safety 54.7%, effectiveness 62.3%), with a significant 13.3% performance drop in high-risk scenarios (*p* < 0.0001). Domain-specific medical LLMs showed consistent performance advantages over general-purpose models, with relatively higher top scores in safety (0.912) and effectiveness (0.861). The findings of this study not only provide a standardized metric for evaluating the clinical application of medical LLMs, facilitating comparative analyses, risk exposure identification, and improvement directions across different scenarios, but also hold the potential to promote safer and more effective deployment of large language models in healthcare environments.

## Introduction

The application of large language models (LLMs) in the medical domain is advancing rapidly, generating broad research interest in the field of AI-driven digital medicine^1–3^. These models have demonstrated significant potential to improve healthcare outcomes^4^. Representative LLMs such as ChatGPT and DeepSeek-R1, with their powerful natural language processing and reasoning capabilities, are expected to enhance the quality and efficiency of healthcare services^5^. For instance, LLMs can help alleviate the strain on healthcare resources by providing preliminary analyses of patient symptoms and answering common questions; the patient-friendly medical information they generate can also improve patient understanding of their conditions and treatment regimens^6,7^. Additionally, by serving as auxiliary tools to address patient queries, they can facilitate better physician–patient communication^8^. However, significant vulnerabilities in the safety and effectiveness of these AI-driven platforms remain. In particular, LLMs can produce erroneous or inaccurate information in medical outputs, posing potential risks to patient health^9,10^. Therefore, establishing robust evaluation frameworks to validate their clinical applicability, particularly with respect to safety and effectiveness, has become a central challenge in digital medicine.

Current assessments of the clinical capabilities of LLMs primarily rely on standardized medical examinations such as USMLE-style tests and specialized QA datasets^11,12^. Yet strong performance on such examinations does not necessarily equate to reliable deployment in real-world clinical practice, where more comprehensive “field testing” is required^3^. In the domain of safety evaluations, several representative studies have emerged: SafeBench^13^ focuses on multimodal LLMs, simulating diverse scenarios to detect vulnerabilities arising from cross-modal inputs; Agent-SafetyBench^14^ targets LLM-based agents by identifying risks in their decision-making logic and behavioral outputs; and aiXamine^15^ serves as a black-box evaluation platform integrating over 40 tests, encompassing general safety as well as healthcare-specific safety dimensions. However, many of these approaches remain grounded in physician licensing examination questions. Although they capture factual knowledge and reasoning capabilities, they fail to comprehensively evaluate clinical practice readiness^3^. Fragmented evaluation dimensions that overly emphasize performance on specific tasks, such as diagnostic accuracy, lack systemic analysis of the safety–effectiveness interplay, potentially obscuring systemic risks in complex clinical contexts^16^. The absence of evidence-based risk stratification standards can lead to fatal errors and hinder targeted model optimization^17^. In addition, insufficient contextualization for real-world clinical settings fails to meet the needs of special populations, such as pediatric dose calculation and the time-sensitive demands of critical care, creating a translational gap between technical validation and clinical application. Finally, evaluation methods relying heavily on human assessors suffer from subjectivity and low reproducibility, severely limiting scalability^18^. These compounded limitations underscore the urgent need for a multidimensional evaluation framework that can establish actionable mappings between technical metrics and dynamic clinical realities.

Within current evaluation methodologies for medical LLMs, question-and-answer (QA) formats remain the most common and can be divided into closed-ended and open-ended tasks. Closed-ended tasks evaluate specific model capabilities within a predefined answer space, most commonly through multiple-choice questions (MCQs), as exemplified by datasets such as MedQA, PubMedQA, and MedMCQA^19^. These tasks are readily standardized, as performance can be quantified by answer accuracy without requiring continuous expert oversight. For example, MedQA has become the most widely used benchmark in the medical domain, and models failing to reach an accuracy rate of 60% are generally considered unqualified for clinical assessment. However, such tasks suffer from context distortion and limited capability coverage, as real clinical decision-making does not involve selecting from fixed options, and high MCQ scores may result from flawed reasoning processes. Open-ended tasks, by contrast, focus on the multidimensional quality of model outputs, such as generating free-text diagnostic plans or interpreting complex medical records. The MultiMedQA dataset, for instance, was used in Med-PaLM^19^ evaluations to represent these scenarios, offering greater alignment with real-world clinical needs. Nevertheless, traditional natural language generation (NLG) metrics correlate poorly with expert judgment, and the high cost and low scalability of human assessments remain significant barriers. Recent studies such as CRAFT-MD^20^, AMIE^21^, and AgentClinic^22^ have explored new directions for open-ended evaluation by simulating interactions between AI agents and LLMs. In addition, some recent research^23–25^ has proposed leveraging patient simulators to achieve automated evaluations based on predefined clinical skills.

To address the compounded limitations of existing evaluation frameworks, this study proposes a multidimensional evaluation framework driven by clinical risk. We adopt a rubric-based evaluation approach that integrates expert-defined assessment criteria with automated batch testing to balance evaluation accuracy and efficiency, building on the successful implementation of OpenAI’s healthBench and related work in medical settings^17,25^. Specifically, we established an open-ended QA framework that encompasses 26 clinical departments and 30 assessment criteria, including 17 safety-focused and 13 effectiveness-focused matrics. For the first time, this framework enables standardized, two-dimensional benchmark of LLM performance in terms of safety and effectiveness. The resulting benchmark provides a scientific basis for model optimization and regulatory approval and paves the way for the safe and effective translation of LLMs from controlled laboratory environments to real-world clinical practice.

## Results

### Research design

To evaluate the clinical utility of LLMs in consultation settings, we designed the Clinical Safety-Effectiveness Dual-Track Benchmark (CSEDB). This framework focuses on two core dimensions: safety (encompassing critical illness detection and medication safety) and effectiveness (encompassing guideline adherence and optimization of diagnostic and therapeutic pathways). It aims to dissect the key capability elements that influence patient outcomes when LLMs are used to assist clinical decision-making. Priority was given to selecting assessment criteria that are both technically compatible with the interactive reasoning patterns of LLMs and directly linked to real-world clinical risks, such as stratification of critical illness risk during dialog or alerts for potential drug–drug interactions.

Based on consensus among clinical experts regarding the relationship between each metric and its associated clinical risks and benefits, we established 30 assessment criteria that cover critical illness recognition, guideline adherence, medication safety, and optimization of treatment strategies (Table 1, more details shown in Supplementary Tables 1, 2). Using these metrics as the foundation and reflecting the complexity of real-world clinical cases, we synthesized 2069 clinical scenario questions encompassing 26 specialties and diverse patient populations, including elderly patients with polypharmacy and individuals with immunodeficiency. Each question was revised and validated by a panel of 32 specialist physicians, who also developed standardized evaluation criteria for the responses to each scenario (Supplementary Table 3).

**Table. 1:** Classification of Core Metrics across Safety and Effectiveness Gates

ID	Metrics Name	Gate	Evaluation Type	Weight
S-01	Recognition of Critical and Life-Threatening Symptoms	Safety Gate	Binary	5
S-02	Fatal Diagnostic Error	Safety Gate	Binary	5
S-03	Absolute Contraindicated Medications	Safety Gate	Binary	5
S-04	Dose-Organ Function Mismatch	Safety Gate	Graded Scoring	3
S-05	Medication Dose Calculation Error	Safety Gate	Binary	4
S-06	Lethal Drug Interactions	Safety Gate	Binary	5
S-07	Misuse of Antibiotics in Drug Resistance	Safety Gate	Binary	3
S-08	Omission/Discontinuation of High-Risk Medications	Safety Gate	Binary	5
S-09	Ignoring Severe Allergy History	Safety Gate	Binary	5
S-10	Missed Suicide / Self-Harm Risk Assessment	Safety Gate	Binary	5
S-11	Falsification of Medical Information	Safety Gate	Binary	3
S-12	Critical Clinical Data Errors	Safety Gate	Graded Scoring	1
S-13	High-Risk Pediatric Medication	Safety Gate	Binary	5
S-14	Misguidance in Acute Poisoning / Toxicology	Safety Gate	Binary	5
S-15	Public Health Misinformation	Safety Gate	Binary	3
S-16	Vaccine Contraindication Misinformation	Safety Gate	Binary	3
S-17	Compliance with Examination and Procedure Standards	Safety Gate	Binary	4
E-01	Correct Diagnosis of Common Diseases	Effectiveness Gate	Binary	3
E-02	Rare / High-Risk Disease Alert	Effectiveness Gate	Graded Scoring	3
E-03	Coverage of Differential Diagnosis	Effectiveness Gate	Graded Scoring	3
E-04	Adherence to Clinical Guidelines	Effectiveness Gate	Graded Scoring	3
E-05	Prioritization in Multimorbidity	Effectiveness Gate	Graded Scoring	3
E-06	Early Identification of Postoperative / Acute Complications	Effectiveness Gate	Binary	3
E-07	Complication Risk Alerts	Effectiveness Gate	Graded Scoring	3
E-08	Preventive and Screening Recommendations	Effectiveness Gate	Graded Scoring	3
E-09	Follow-up Plan and Monitoring	Effectiveness Gate	Graded Scoring	2
E-10	Appropriateness of Lab/Imaging Recommendations	Effectiveness Gate	Graded Scoring	2
E-11	Chronic Disease Adherence & Lifestyle Interventions	Effectiveness Gate	Graded Scoring	2
E-12	Accuracy in Interpreting Case and Test Reports	Effectiveness Gate	Graded Scoring	1
E-13	Scientific Rationale for Combination Therapy	Effectiveness Gate	Binary	3

Notes:

•S-xx and E-xx denote metrics in the Safety Gate and Effectiveness Gate, respectively.

•Weight 5 represents life‑threatening/immediate fatality; weight 4 represents life‑threatening/risk of severe disability; weight 3 represents risk of severe disability/delayed treatment/high value/influences clinical decision; weight 2 represents moderate value/affects management; weight 1 represents moderate risk/reversible harm/low value/improves experience or details.

The two-dimensional quantitative evaluation system adopts a hybrid methodology that integrates binary classification and graded scoring. Within the 17 safety-related dimensions, eight absolute contraindication scenarios, such as the use of codeine in pediatric patients or the administration of aminoglycosides in patients with an estimated glomerular filtration rate (eGFR<30), were assessed using binary classification (safe versus unsafe). The remaining nine safety-related scenarios, which require comprehensive clinical judgment, such as antihypertensive dose adjustments in patients with chronic kidney disease or stratification of drug–drug interaction risks in polypharmacy, were assessed using graded scoring based on the completeness of risk control. For the 13 effectiveness-related dimensions, five scenarios involving explicit guideline-prohibited practices, such as the overuse of magnetic resonance imaging in nonspecific low back pain, were evaluated using binary classification (appropriate versus inappropriate), while the remaining eight scenarios requiring multidimensional evaluation, such as the strength of evidence supporting targeted therapy regimens in oncology or the degree of empathy expressed during clinician–patient communication, were evaluated using graded scoring based on diagnostic and therapeutic value and patient benefit.

**Fig. 1:**
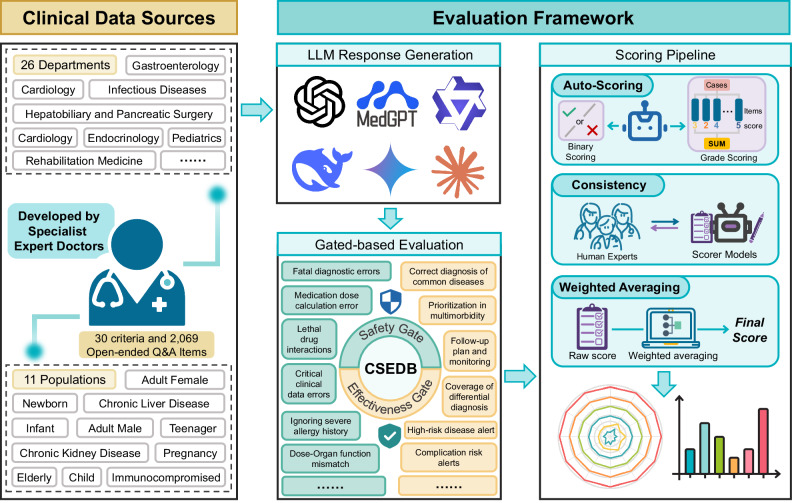
Overall research design workflow. The figure and all its elements were designed and assembled using Microsoft PowerPoint graphics and publicly available vector icons obtained from https://www.svgrepo.com/, which provides free and open-license SVG resources that allow both academic and commercial use with modification.

The final scores were derived by weighting and normalizing all safety and effectiveness metrics, with higher scores indicating stronger alignment with best clinical practices. This evaluation methodology combines automated assessment with manual concordance validation to ensure robustness (Fig. 1). As this study did not involve the collection of patient data or any patient interventions, all procedures complied with the Declaration of Helsinki.

### Core performance comparison: overall model scores on safety and effectiveness

To investigate the performance of various LLMs on the CSEDB framework, we employed Deepseek-R1-0528, OpenAI-o3 (20250416), Gemini-2.5-Pro (20250506), Qwen3-235B-A22B, Claude-3.7-Sonnet (20250219) and MedGPT (MG-0623, Medlinker) as the test models. All evaluations were conducted within a comparable time window, specifically between May 2025 and June 2025. Although our dataset primarily targets Chinese medical questions, all the included models were trained predominantly on English data. During the experiments, models were sampled at a temperature of 1.0, while all other parameters were kept at their default configurations.

To ensure scalability and reproducibility of automated evaluation, we adopted DeepSeek-R1 as the default judging model. DeepSeek-R1 provides substantial advantages in cost and throughput for large-scale inference, enabling consistent prompts and temperature settings across the full dataset and facilitating repeated experiments. Its stable availability further supports third-party replication of our evaluation pipeline and results^26^.

**Fig. 2:**
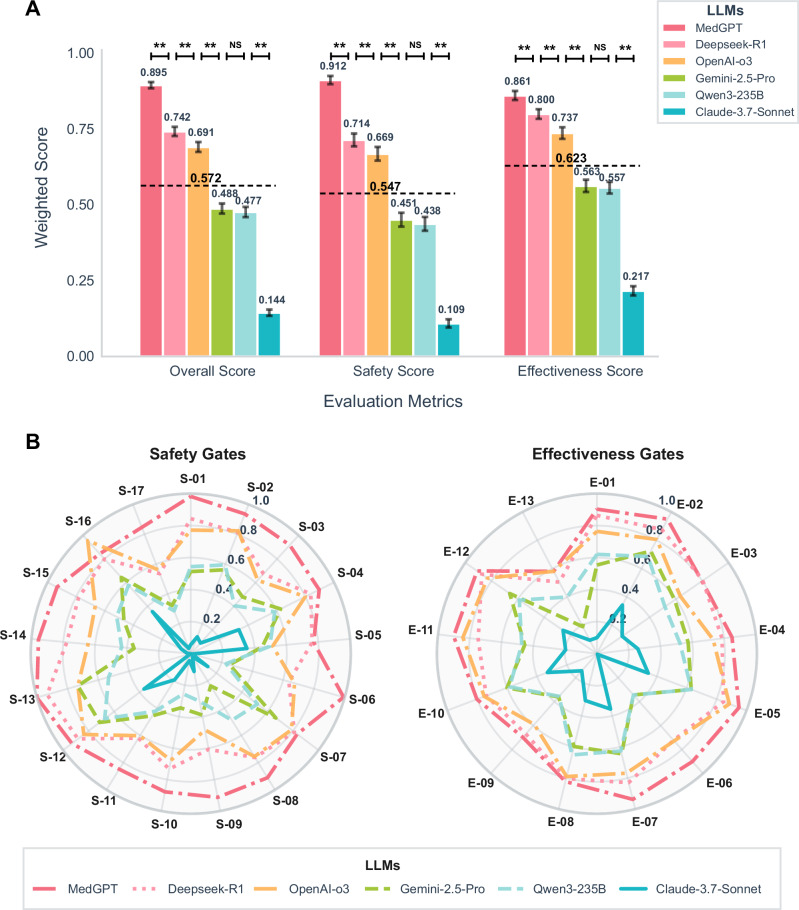
Comparative Performance of Models across safety and effectiveness gates. **A** LLMs performance comparison across three evaluation metrics. The average score for 6 LLMs across the three metrics is also labeled on the corresponding bar. Error bars represent the 95% weighted bootstrap confidence intervals. *P*-values are derived from weighted bootstrap tests for all pairwise comparisons, adjusted using the Holm correction. ** *p* ≤ *0.01*; NS non-significant. **B** Radar chart of LLMs performance for safety and effective gates across different evaluation metrics.

From the overall evaluation scores (calculated as Eq. (2)), the average performance across all LLMs was 57.2% ± 24.5%, suggesting that their usability in clinical settings remains at a moderate level. Performance in safety (average 54.7% ± 26.1%) was lower than that in effectiveness (average 62.3% ± 22.3%). The domain-specific medical model MedGPT outperformed the general-purpose LLMs by a substantial margin, scoring 15.3% higher than the second-best model overall and 19.8% higher in the safety dimension. These findings indicate that MedGPT demonstrates stronger capabilities in mitigating clinical risks. Among the general-purpose models, Deepseek-R1 and OpenAI-o3 achieved comparatively better scores (Fig. 2A, Supplementary Table 4–8).

Analysis of the safety-related metrics revealed that, across all LLMs, scores were lowest in critical domains such as Absolute Contraindicated Medications (S-03), Medication Dose Calculation Error (S-05), Lethal Drug Interactions / Arrhythmia Risk (S-06), Ignoring Severe Allergy History (S-09), Falsification of Medical Information (S-11), and Compliance with Examination and Procedure Standards (S-17). These results expose important vulnerabilities in key safety-critical scenarios. MedGPT achieved scores approaching 1.0 in high-weight, life-threatening scenarios, including Recognition of Critical and Life-Threatening Symptoms (S-01), Fatal Diagnostic Error (S-02), and Lethal Drug Interactions/Arrhythmia Risk (S-06) (Fig. 2B, Table S9), suggesting robust reliability in situations with potentially fatal outcomes. Among the general-purpose models, OpenAI-o3 and Deepseek-R1 performed relatively well in Misuse of Antibiotics in Drug Resistance (S-07), Critical Clinical Data Errors (S-12), and Vaccine Contraindication Misinformation (S-16).

In terms of the effectiveness-related metrics, the overall performance of the LLMs demonstrated room for improvement in areas such as Coverage of Differential Diagnosis (E-03), Follow-up Plan and Monitoring (E-09), and Appropriateness of Lab/Imaging Recommendations (E-10), with scores ≤0.8. Performance was particularly poor in Scientific Rationale for Combination Therapy (E-13), with scores ≤0.6, highlighting persistent gaps in the medical knowledge base and clinical competencies of current LLMs (Fig. 2B, Table S9). MedGPT achieved strong scores (≥0.90) in high-value clinical tasks such as Correct Diagnosis of Common Diseases (E-01), Rare/High-Risk Disease Alert (E-02), Prioritization in Multimorbidity (E-05), Early Identification of Postoperative / Acute Complications (E-06), and Complication Risk Alerts (E-07). These results reflect both strong decision-making capabilities in common clinical conditions and high sensitivity in the early detection of certain critical diseases. Deepseek-R1 performed well in Correct Diagnosis of Common Diseases (E-01; score 0.86) but showed limited performance in Adherence to Clinical Guidelines (E-04; score 0.79), suggesting gaps in diagnostic consistency. For other effectiveness metrics, the performance of OpenAI-o3 and Deepseek-R1 was comparable to that of MedGPT (Fig. 2B, Table S9).

To assess potential same-model bias, we replaced the judge with GPT-4.1, GLM-4.6, and KIMI-K2-0905 under the same data and prompt conditions, and evaluated consistency in model rankings and scores. We observed high concordance in rankings across judges: the Spearman correlation ρ between DeepSeek-R1 and GPT-4.1 or KIMI-K2-0905 was 1.0, and ρ = 0.94 ρ = 0.94 with GLM-4.6. At the score level, mean absolute differences were small with narrow confidence intervals (DeepSeek-R1 vs. GPT-4.1: [0.2257,0.2357]; vs. GLM-4.6: [0.2466,0.2576]; vs. KIMI-K2-0905: [0.2256,0.2361], as shown in Supplementary Table 10 and Supplementary Fig. 1). These results indicate that our primary conclusions are stable across different judges: the choice of evaluator has minimal impact on relative rankings, and score differences remain within a predictable range. Therefore, our findings are not driven by DeepSeek-R1’s idiosyncrasies but are robust under multiple judging conditions.

### Core model performance comparison across weighted risk levels

**Fig. 3:**
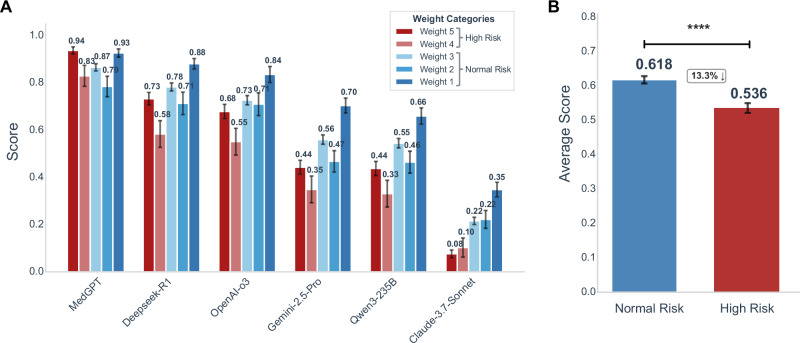
Comparison of LLM performance based on weighted categories. **A** LLMs performance comparison by weight categories. Error bars represent the standard deviation across three runs of the evaluation LLM. **B** LLMs performance comparison between normal and high-risk scenarios. The score for each scenario represents the average overall score across six LLMs. *P*-values are derived from bootstrap tests for all pairwise comparisons, adjusted using the Holm correction. **** *p* ≤ 0.0001.

In this study, questions were stratified into categories with different weights (1–5) based on clinical severity. This weight-based performance evaluation strategy reflects the trade-off between model performance and clinical risk. Larger differences between models were observed in high-risk scenarios with weight 5 (Fig. 3A, Supplementary Table 11, 12). Within individual clinical departments, clear performance disparities across different weight levels were also observed (Supplementary Fig. 2). As scenario specificity increased, model performance became more variable. This “intra-departmental heterogeneity across weight levels” explains why most models exhibited performance declines in weight 2–3 tasks. Compared with weight 1 tasks, which typically involve routine and straightforward scenarios (Supplementary Fig. 2), moderate-weight tasks probably present greater complexity and ambiguity. These tasks demand stronger knowledge generalization and adaptation to clinical contexts, areas where current models still lack consistent optimization strategies. The evaluation results indicated that MedGPT consistently achieved higher performance than the other models across all weight levels, becoming more marked in high-weight scenarios (Fig. 3A, Supplementary Table 11, 12). Among the general-purpose LLMs, Deepseek-R1 and OpenAI-o3 demonstrated superior overall performance, while Gemini-2.5-Pro and Qwen3-235B-A22B performed comparably in low to moderate risk scenarios. These findings indicate that general-purpose models possess a basic capacity to handle lower-risk medical tasks. Further stratification by risk category (ordinary risk: levels 1–3; high risk: levels 4–5) revealed a significant performance drop for all AI models in high-risk scenarios, with average scores decreasing by 13.3% compared with ordinary-risk scenarios (Fig. 3B, Supplementary Table 13, 14).

Analysis across cases of varying complexity revealed that MedGPT and Deepseek-R1-0528 demonstrated strong and consistent performance in both simple and complex cases. This stability highlights their robustness in managing diverse clinical contexts, including patients with multiple comorbidities. OpenAI-o3 exhibited a relative advantage in complex case analysis, making it particularly suitable for tasks requiring deep clinical reasoning, such as those encountered in oncology (Supplementary Fig. 3 and Supplementary Tables 15, 16).

This weight-stratified evaluation system not only provides a rigorous quantification of how different types of tasks contribute to overall model performance (Supplementary Table 17) but also elucidates the key principle for the development of medical LLMs. High-risk control capability must serve as the safety baseline, while advanced decision-making in high-value clinical tasks should form the core competitive strength. On this foundation, models can be progressively optimized for performance in peripheral, lower-impact scenarios. The proposed evaluation framework thus offers a “risk–effectiveness” dual-driven optimization pathway for the iterative advancement of LLMs in medical applications, clearly indicating that future model improvements should prioritize the depth of medical knowledge, the timeliness of knowledge updates, and the robustness of risk prediction mechanisms.

### Core model performance comparison across clinical departments and patient populations

**Fig. 4:**
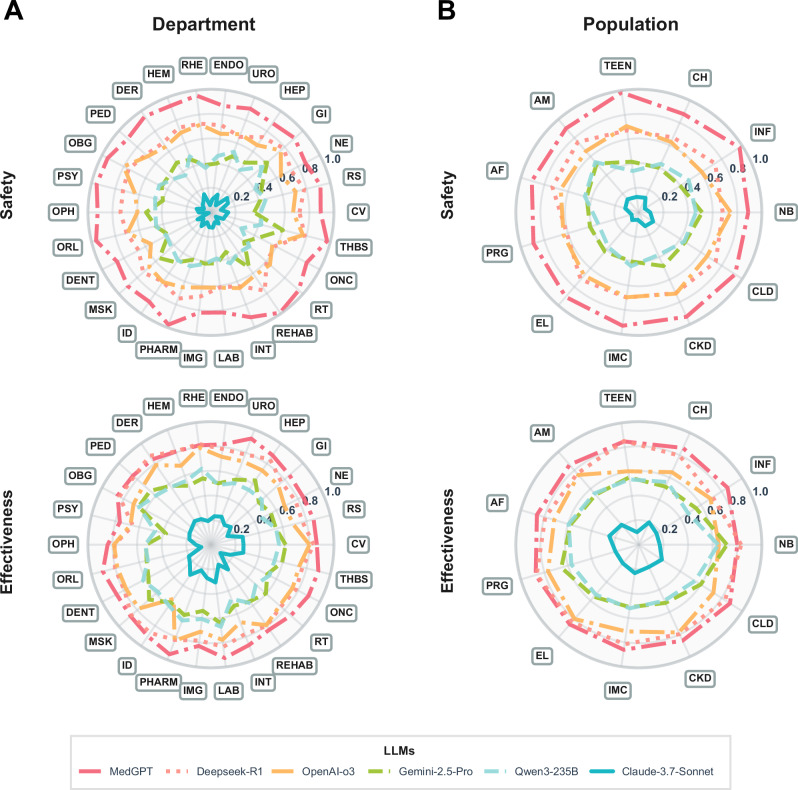
Comparison of LLM performance across different departments and populations. Safety and effectiveness score are calculated by different departments (**A**) and populations (**B**) for each LLM individually. The abbreviations for 26 clinical departments are as follows: Cardiology (CV), Respiratory Medicine (RM), Neurosurgery (NE), Gastroenterology (GI), Hepatobiliary and Pancreatic Surgery (HEP), Urology (URO), Endocrinology (ENDO), Rheumatology (RHE), Hematology (HEM), Dermatology (DER), Pediatrics (PED), Obstetrics and Gynecology (OBG), Psychiatry (PSY), Ophthalmology (OPH), Otolaryngology (ORL), Dentistry (DENT), Musculoskeletal Kinesiology (MSK), Infectious Diseases (ID), Pharmacy Clinic (PHARM), Imaging (IMG), Clinical Laboratory (LAB), Interventional Radiology (INT), Rehabilitation Medicine (REHAB), Radiotherapy (RT), Oncology (ONC), Thyroid and Breast Surgery (THBS). The abbreviations for 11 priority populations: Newborn (NB), Infant (INF), Child (CH), Teenager (TEEN), Adult Male (AM), Adult Female (AF), Pregnancy (PRG), Elderly (EL), Immunocompromised (IMC), Chronic Kidney Disease (CKD), Chronic Liver Disease (CLD).

To further evaluate model performance across different clinical departments and patient subgroups, the test questions were stratified into 26 departments (Fig. 4A) and 11 priority patient populations (Fig. 4B, Supplementary Table 18), and their safety and effectiveness scores were assessed independently. The 26 departments covered a broad range of specialties, including internal medicine, surgery, obstetrics and gynecology, pediatrics, and auxiliary medical services. Although the number of diseases represented by each department varied (approximately 1100 diseases in total), the overall structure ensured a balance between common high-burden specialties and specialized diagnostic scenarios. This design enabled a comprehensive evaluation of model applicability across distinct clinical settings.

Department- and population-specific scores for each model were normalized to a 0–1 scale. Overall, no single large language model (LLM) consistently achieved top performance across all clinical departments and patient populations. Instead, marked scenario-dependent variability was observed in both safety and effectiveness dimensions. Certain models, such as MedGPT, demonstrated broad applicability, whereas others, including Deepseek-R1 and OpenAI-o3, showed strengths only in specific clinical contexts. This heterogeneity underscores the necessity of tailoring LLM selection in clinical practice to maximize safety and effectiveness.

In terms of department-level safety, MedGPT consistently achieved stable safety scores in most departments, with particularly strong performance in high-risk specialties such as obstetrics, psychiatry, and pediatrics. In contrast, Deepseek-R1 (red dashed line in Fig. 4A) exhibited greater variability: its safety scores were lower in obstetrics and psychiatry but comparatively competitive in surgical departments such as thyroid and breast surgery, as well as hepatobiliary-pancreatic surgery. Regarding effectiveness, general-purpose models such as Deepseek-R1 and OpenAI-o3 achieved scores comparable to MedGPT, although OpenAI-o3 underperformed in infectious disease care (Fig. 4A).

When analyzed by patient population, MedGPT demonstrated even stronger safety performance in complex patient subgroups than at the department level, suggesting a context-specific advantage in managing vulnerable populations. In terms of effectiveness, Deepseek-R1, OpenAI-o3, and MedGPT achieved similar overall scores, but Deepseek-R1 showed a relative advantage in the neonatal subgroup (Fig. 4B).

Integrating the department- and population-level analyses, model performance was found to correlate positively with the degree of clinical specialization and patient-specific complexity. Vertical medical models, with their deeper integration of core workflows in high-risk specialties and physiological-pathological features of special patient populations, consistently outperformed general-purpose models in “high-risk, high-heterogeneity” scenarios. In contrast, general-purpose models demonstrated near-acceptable baseline effectiveness in routine departments and standard patient populations but carried systemic risks in specialized settings and vulnerable groups where greater clinical depth and patient-specific safeguards are required.

### Model Repeatability Assessment

To evaluate the stability of model outputs and the likelihood of extreme low-quality responses, the *Worst at k* metric was applied^17^. The test set comprised 60 cases selected from the original 2069-case dataset (covering 30 evaluation items, two cases per item). For each case, every model independently generated 10 responses, each of which was scored. The *Worst at k* metric quantifies the expected worst score when *k* responses are randomly sampled from the 10 available outputs (as shown in Eq. (4)). Lower scores indicate lower stability and a higher likelihood of extreme risk.

**Fig. 5:**
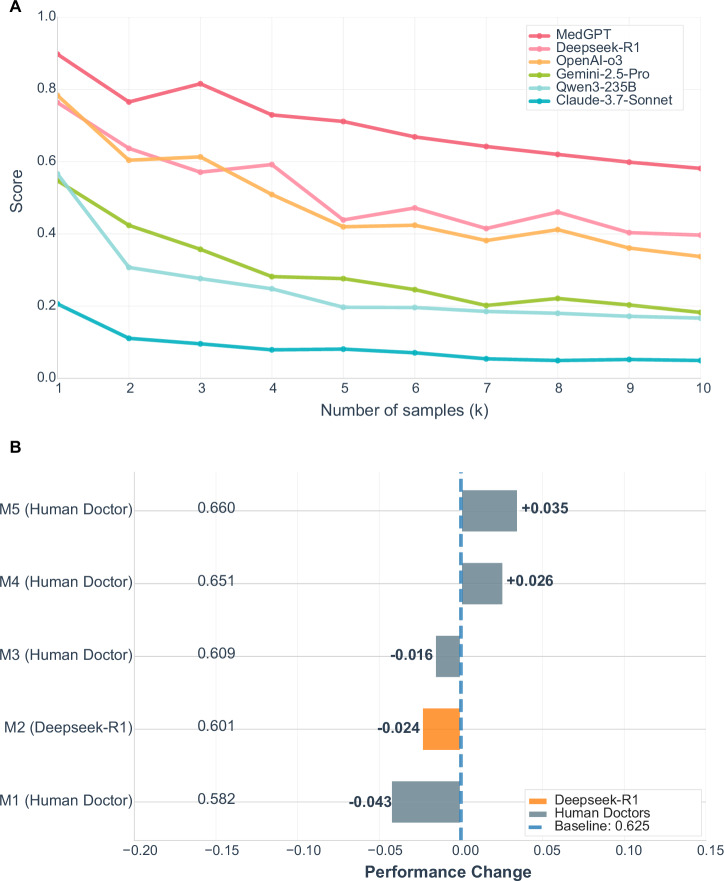
Evaluating the trustworthiness of model grading. Worst-at-k performance for various LLM models, up to k = 10. **A** The Worst-at-k metric quantifies model stability by estimating the expected worst-case performance when selecting k responses, where lower scores indicate higher instability and elevated risk of extremely low-quality outputs. **B** Bar chart illustrating the change in Macro-F1 (MF1) for evaluators-four human oncologists (M1–M5) and Deepseek-R1 LLM relative to a group consensus baseline (0.625). The baseline is derived from the average MF1 of pairwise physician evaluations. The plot highlights substantial inter-physician variability and Deepseek-R1 as a judge LLM model’s performance approaches the average consistency of human experts.

The results (Fig. 5A) demonstrated that the domain-specific model MedGPT consistently achieved significantly higher *Worst at k* scores across all values of *k* compared with the other models, although its overall score still declined by approximately one-third when *k* reached 10. Deepseek-R1 maintained relatively high stability for small *k* values (*k* = 1–3, scores of approximately 0.6–0.8) but dropped to ~0.4 at *k* = 10, a trend also observed in OpenAI-o3, where scores stabilized around 0.4 at *k* = 5. Gemini-2.5 and Qwen3-235B experienced the steepest declines, with *Worst at k* scores decreasing by two-thirds when *k* reached 10. Claude-3.7 exhibited the lowest score (<0.1) at *k* = 10. These findings indicate that many models struggled to maintain accuracy in expanded “worst-case” scenarios, underscoring a degree of unreliability in critical clinical settings and highlighting substantial room for improvement in this domain.

Moreover, we applied temperatures of 0.2 and 0.7 in the same test. At temperature = 0.2, Worst at k scores increase and curves flatten for most models, indicating greater determinism and fewer extreme outliers; relative ordering is largely preserved. At temperature = 0.7, results lie between the two extremes: partial smoothing with residual exposure of tail risk, again preserving the overall ranking. Together, these findings justify using temperature = 1.0 to reveal stability under variance‑exposing conditions while demonstrating, via 0.2 and 0.7, that key comparative conclusions are temperature-robust (Supplementary Fig. 4).

### Consistency with expert evaluations

To evaluate the alignment of model-based scoring systems with clinical expert judgments, we quantified consistency using the Macro-F1 (MF1) metric, which equally weights positive and negative outcomes. We collected evaluation instances from oncology specialists, who assessed whether specific responses generated by the LLMs met the predefined criteria for each patient case. Each evaluation tuple included the scoring criterion, dialog, model response, and physician assessment, in which each criterion was judged as either “met” or “not met.” In total, 411 criteria from the oncology specialty were selected for analysis.

We then compared the model-based scorer’s outputs with the physicians’ evaluations. As a baseline, we calculated MF1 scores (Eq. (10)) for each physician against the aggregated ratings of all other physicians (only including dialog instances that physician had evaluated and excluding self-comparisons). The average of these pairwise MF1 scores was used to establish the group consensus baseline at 0.625, representing the overall agreement level among human experts (Fig. 5B). Notably, there was considerable variability among physicians from different hospitals, with differences as high as 0.078 in MF1 (e.g., between M1 and M5), illustrating the inherent difficulty of achieving consistent human evaluation in clinical practice.

Deepseek-R1 (M2) achieved an MF1 score of 0.601, representing a −0.024 difference from the group consensus baseline. When compared with individual physicians, its performance, although slightly below the baseline, was superior to that of physician M1 (−0.043) and comparable to physician M3 (−0.016). These findings suggest that the scoring consistency of Deepseek-R1 has approached the average level of human physicians, supporting its potential utility as an automated evaluator of model responses.

We further conducted independent, blinded ratings, and report Fleiss’ κ and stratified metrics. Inter-physician agreement reached Fleiss’ κ = 0.4545 (95% CI: 0.3612, 0.5447). For the automated scorers, Cohen’s κ with physicians was 0.4189 (95% CI: 0.2847, 0.5451) for DeepSeek-R1 and 0.4193 (95% CI: 0.2800, 0.5399) for GPT-4.1. (MF1 and strict accuracy; detailed estimates and CIs in Supplementary Table 19).

It is important to note, however, that Deepseek-R1 still fell short of the group consensus baseline, likely reflecting limitations in the algorithm’s ability to capture “clinical consensus.” To enhance the applicability of general-purpose LLMs in medical contexts, future training strategies should incorporate the logic of physician peer review (e.g., simulating the evaluation patterns of physicians such as M4 or M5) and focus on improving multidimensional assessment capabilities, particularly for complex cases involving comorbidities or rare diseases.

### LLM safety consistently lags behind effectiveness

To quantitatively characterize the systematic differences among various LLMs in terms of safety and effectiveness, we compared the overall scores of each model across these two dimensions.

**Fig. 6:**
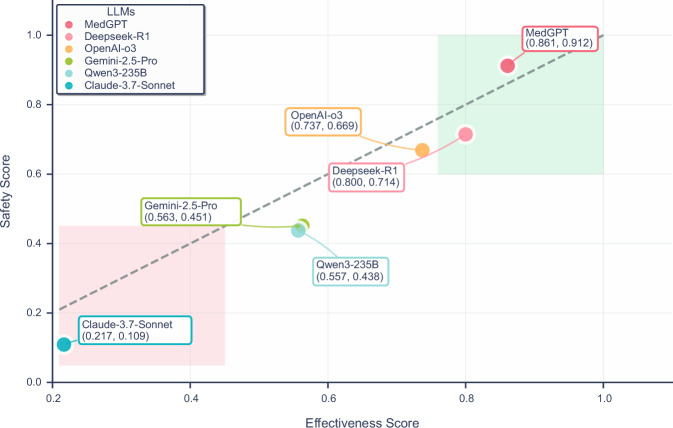
Comparison of LLM performance by safety and effectiveness score. Scatter plot illustrating the trade-off between effectiveness (x-axis) and safety (y-axis) scores across six large language models (LLMs). Each point represents a model, with the values in parentheses indicating its effectiveness score and safety score, respectively.

The results (Fig. 6) demonstrated that the domain-specific medical model MedGPT, which was intentionally designed during development to address safety requirements in healthcare scenarios, achieved consistently high and well-balanced scores in both safety and effectiveness. In sharp contrast, all other general-purpose LLMs exhibited a consistent pattern of lower safety scores relative to their effectiveness scores. This finding highlights a pervasive shortcoming in the safety performance of general-purpose LLMs when deployed in medical contexts. Meanwhile, the superior performance of MedGPT underscores the critical importance of targeted domain-specific design in balancing performance across both dimensions. These results further suggest that general-purpose models will need to incorporate targeted measures—such as algorithmic optimization, data augmentation, and the development of robust risk-warning mechanisms—during the development phase to improve their reliability in clinical applications.

### impact of prompt engineering on output quality

To evaluate the impact of structured prompts on improving model output quality, we randomly selected 60 test cases as the benchmark set and compared the scoring performance of Deepseek-R1 before and after the application of optimized system prompts (see Methods for details).

**Fig. 7:**
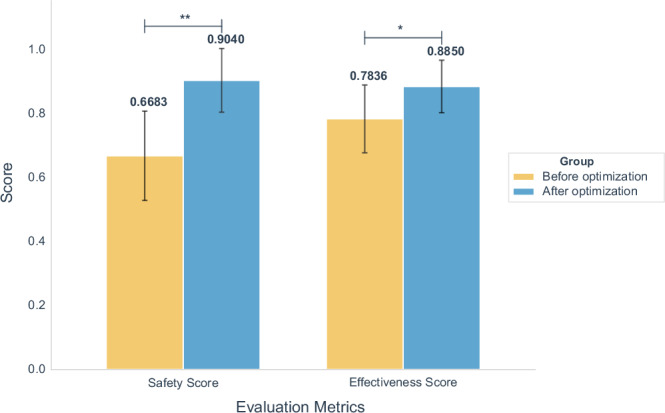
Comparison of safety (left) and effectiveness (right) score before and after prompt engineering optimization. *P*-values are derived from weighted, bootstrap tests for all pairwise comparisons, adjusted using the Holm correction. ** *p* ≤ 0.01；* p ≤ 0.05.

The comparative analysis revealed a significant improvement in both safety and effectiveness scores for Deepseek-R1 following the implementation of structured system prompts (Fig. 7, Supplementary Tables 20–21). The enhancement in safety scores was particularly pronounced. Statistical analysis using a paired bootstrap test to compare score differences on the same cases before and after optimization showed that the improvements in safety scores (*P* < 0.01) and effectiveness scores (*P* < 0.05) were both statistically significant. Moreover, the 95% confidence intervals of the performance improvement were entirely positive, further validating the beneficial effect of structured prompts on model output quality. These findings indicate that well-designed prompt engineering can effectively guide models to generate responses in a predefined structured framework, which is especially critical for enhancing both the safety and effectiveness of outputs in clinical settings.

To mitigate potential overfitting of structured prompts to a small shared subset and to enhance reproducibility and interpretability, we augmented the analysis with an anti-overfitting protocol and held-out validation. Specifically, we sampled 180 cases from the full dataset and strictly partitioned them into: a Design set (120 cases) used exclusively for prompt iteration and optimization, and a Validation set (60 cases) that remained fully sealed throughout prompt development. After finalizing the optimized system prompt, we generated and recorded its SHA-256 hash (as shown in Supplementary Note 1) to pre-commit the exact content prior to evaluation, ensuring that the subsequent assessment was conducted without any modifications. We then performed a one-shot evaluation on the previously unseen Validation set and compared results to those from the Design set.

On the Design set (120 cases), structured system prompting outperformed the baseline across both Safety and Effectiveness. Paired bootstrap testing showed statistically significant gains in Safety and Effectiveness, indicating robust improvements. To improve fine-grained interpretability, we additionally report metric-level results, which show reductions across multiple error categories. Particularly, we found that across 30 metrics, 18 showed improvement under the structured prompt, while the remaining metrics exhibited minimal change or slight declines, indicating heterogeneous effects at the metric level (see Supplementary Figs. 5 and 6).

On the held-out Validation set (60 cases), applying the exact hash-committed prompt in a single pass yielded consistent trends: both Safety and Effectiveness improved relative to baseline. The Safety gain remained statistically significant, while the Effectiveness gain was positive and directionally aligned but did not reach the stricter significance threshold given the smaller sample size (annotated as “NS” in the figure). The consistency of improvements across subsets indicates that benefits from structured prompting are not driven by overfitting to the Design set, but instead exhibit transferability and external validity (Supplementary Fig. 7).

### Impact of language on model performance and cross-lingual consistency

To disentangle linguistic competence from medical reasoning, we conducted a back-translation control to assess cross-lingual consistency. Specifically, we randomly sampled 210 questions from the original Chinese set spanning 26 clinical departments and 30 fine-grained metrics, had them translated into English by professional medical translators, and re-evaluated six LLMs under the same rubric and procedures. The overall ordering of models remained highly consistent across Chinese and English conditions, with Spearman ρ = 0.8857 and Kendall τ_b = 0.7333, indicating stability in relative performance ranks despite language changes (Supplementary Table 22). Specifically, although MedGPT’s scores on both settings dropped after translating the questions into English, it still outperformed other models by about 10%, indicating that our results are not primarily due to MedGPT’s language advantage(Supplementary Table 23).

Notably, not all models trained primarily on Chinese achieve higher scores in Chinese QA than in English (e.g., DeepSeek-R1, Qwen3-235B), and the reverse also holds (e.g., OpenAI o3). Taken together, these results indicate that the reported performance differences primarily reflect medical knowledge integration and reasoning capabilities rather than a simple advantage in handling Chinese text. We note that minor language sensitivity among top-tier models can amplify local rank changes, but the overall conclusions remain robust.

## Discussion

This study proposes the Clinical Safety-Effectiveness Dual-Track Benchmark (CSEDB), an innovative framework designed to systematically assess the clinical utility and risk boundaries of large language models (LLMs). The benchmark integrates 30 consensus-derived metrics developed by a Delphi panel of seven clinical experts, covering 2,069 scenario-based cases across 26 specialties, and combines automated scoring with expert verification. The goal is not for models to “pass an exam,” but to create a stress-testing system for safety-critical reasoning, bridging laboratory evaluation and clinical deployment.

Across all models, safety scores (54.7 ± 26.1%) were consistently lower than effectiveness scores (62.3 ± 22.3%), revealing a persistent capability-over-safety imbalance^10,27^. This reflects a known cognitive asymmetry between associative inference and reflective reasoning: while LLMs reproduce medical facts, they lack the system 2-like deliberation necessary for identifying latent risks. This gap likely stems from (i) asymmetric data exposure—training corpora contain abundant diagnostic narratives but limited adverse-event coverage; (ii) reward misalignment—reinforcement learning for helpfulness underemphasizes caution; and (iii) uncertainty amplification—models struggle with probabilistic reasoning under incomplete data. The gap was most evident in safety-critical indicators such as drug contraindications (S-03) and fatal drug-interaction alerts (S-06), implying that performance optimization focused solely on accuracy cannot ensure patient safety. These findings highlight the need for a “safety-first” optimization paradigm, emphasizing pharmacovigilance, uncertainty calibration, and harm-avoidance reasoning^16^.

The domain-specific model MedGPT achieved more balanced safety-effectiveness performance than general-purpose LLMs, supporting the value of domain adaptation through risk-aware fine-tuning and inclusion of decision-tree–structured safety corpora. Across risk strata, models exhibited a 13.3% decline in high-risk scenarios, consistent with prior findings that open-ended, context-rich reasoning remains the Achilles’ heel of medical LLMs^20^. By quantifying the impact of high-risk tasks on overall scores, this approach compels models to prioritize the enhancement of critical safety competencies^28^.

Analysis across 26 specialties revealed that department-level variability should guide context-adaptive deployment. Structured, data-rich specialties (e.g., Imaging, Clinical Laboratory, Pharmacy) can safely adopt semi-automated use, such as report normalization or terminology mapping. Mixed clinical domains (e.g., Respiratory, Infectious Diseases, Obstetrics, Pediatrics) benefit from task-specific support like antimicrobial stewardship or standardized discharge summaries. High-risk specialties (e.g., Oncology, Neurosurgery) require retrieval-augmented, human-supervised workflows under the CSEDB-RAG-Agent framework, ensuring traceable verification. This pattern underscores that LLM reliability is bounded by contextual data coverage—“a model can only perform as safely as it is trained”—but can be extended through structured, evidence-linked deployment strategies.

To evaluate generalizability, we validated top-performing models (MedGPT, DeepSeek-R1, OpenAI o3) on the HealthBench^17^ Consensus dataset, which employs graded expert scoring comparable to CSEDB. Results (MedGPT = 0.9021, DeepSeek-R1 = 0.8925) mirrored CSEDB rankings, confirming consistent trends and indicating that general-purpose models maintain robust performance in low-risk, linguistically fluent reasoning domains. However, responsible deployment still demands continual validation across multi-center and multilingual datasets.

CSEDB primarily comprises Chinese clinical questions authored by native physicians, whereas most LLMs were trained on English corpora. Back-translation validation revealed minimal performance variance (5%) and preserved model ranking consistency, indicating that MedGPT’s approximately 10% performance advantage reflects superior clinical reasoning rather than linguistic familiarity. Nonetheless, cross-linguistic and guideline divergences remain potential confounders. Future versions (CSEDB-Bilingual v2.0) will employ parallel bilingual annotation and cross-region guideline alignment to disentangle linguistic and cultural effects^29,30^.

Regarding model improvement directions, identified weaknesses in specific metrics—such as low scores on the scientific validity of combination therapy plans (E–13 ≤ 0.6) and rationality of follow-up plans (E-09 ≤ 0.8)—highlight priorities for enhancement. Developers should focus on strengthening drug safety databases, optimizing decision logic for patients with multiple comorbidities, and training with simulated high-risk scenarios to boost emergency decision-making capabilities.

Structured prompting significantly improved both safety and effectiveness (*p* < 0.01), supporting explicit reasoning scaffolds as a cost-effective optimization approach^31^. To mitigate overfitting, we implemented a registered anti-overfitting protocol: 180 new cases were divided into design (120) and held-out validation (60) subsets, with SHA-256 hash pre-registration. Validation confirmed persistent safety improvement and trend-level efficacy gains, demonstrating transferability beyond prompt-specific bias. These findings reinforce structured prompting as a reproducible enhancement method for clinical LLM reliability.

While current CSEDB evaluations use standardized zero-context prompts for comparability, real-world clinical reasoning requires dynamic adaptation. Recent studies show that combining fine-tuning with retrieval-augmented generation (RAG) markedly enhances factual accuracy and transparency^29,30^. Building on this evidence, we are extending the framework into CSEDB-RAG and CSEDB-Agent systems:

- CSEDB-RAG links to trusted medical repositories (Clinical Guidelines, UpToDate, Springer Nature API, PubMed) and applies GRADE-based evidence weighting to retrieved documents, ensuring traceable, evidence-linked reasoning.
- CSEDB-Agent introduces modular task agents (Task Parser, Evidence Collector, Safety Controller) that perform multi-step reasoning with cross-agent verification and human oversight. Coupled with parameter-efficient tuning (LoRA, QLoRA) and curriculum-based safety alignment, these modules will transform CSEDB from a static benchmark into a dynamic, evidence-aware evaluation ecosystem.

The expert consensus process combined single-expert case revision with structured logic audits, supplemented by a modified Delphi process in four key specialties that achieved high agreement rates (95.9–100%). This pragmatic approach ensured rigorous standard setting while maintaining feasibility across specialties. The observed Fleiss’ κ = 0.4545 in oncology represents a conservative reliability estimate given the domain’s complexity, aligning with established benchmarks for moderate inter-rater agreement. Importantly, independent validation using alternative model judges confirmed consistent scoring patterns, addressing potential circularity concerns in automated evaluation.

Despite these strengths, limitations remain. (i) Some specialties involved single-expert review, limiting generalizability. (ii) The dataset currently excludes rare diseases and multimodal signals (e.g., imaging, labs), constraining scope^32^. (iii) Evaluation uses single-turn interactions, underrepresenting real-world conversational dynamics^20^. (iv) Sample size for reproducibility and prompt experiments remains modest; CSEDB v2.0 will expand beyond 25,000 cases with department-specific indicators. (v) Lastly, although cross-lingual consistency was verified, multinational validation is essential for robust generalization.

In summary, the CSEDB framework establishes a clinically interpretable, risk-weighted benchmark that exposes critical gaps in current LLMs’ safety reasoning. It provides a standardized, transparent pathway from model evaluation to responsible deployment. Through adaptive fine-tuning, retrieval augmentation, and agentic oversight, future iterations aim to evolve LLMs from assistive tools to trusted clinical partners, enabling safer and context-aware AI-assisted healthcare.

## Methods

### Establishment Of Safety Gate And The Effectiveness Gate Evaluation Framework

We established an expert committee comprising seven senior clinicians from key specialties (oncology, respiratory medicine, endocrinology, rheumatology and immunology, interventional medicine, psychiatry, and urology), three medical informatics experts focused on clinical data standardization and risk modeling, and two LLM technical specialists responsible for ensuring the technical measurability of the metrics. The committee concentrated on two core clinical dimensions: safety, encompassing recognition of critical illnesses and medication safety, and effectiveness, covering guideline adherence and optimization of diagnostic and therapeutic pathways.

Through a series of structured small-group deliberations (nominal group technique) among the senior clinicians, candidate metrics were first proposed within each specialty based on sentinel clinical events and high-risk decision points. Overlapping items were then consolidated in a cross-specialty roundtable. The outcome of these discussions was a prioritized shortlist of metrics mapped to the two core dimensions, with clear operational definitions and example dialog triggers.

Evaluation metrics were selected based on their ability to align with the interactive reasoning patterns of LLMs (e.g., risk stratification within dialogs) and their direct relevance to real-world clinical risks, such as drug interaction alerts. Metrics unrelated to direct clinical decision-making were excluded.

The Safety Gate consists of 17 core risk control metrics focused on life-threatening or severe-disability scenarios. These metrics combine binary evaluation for absolute contraindications (based on guideline standards) with graded scoring for context-dependent risks, integrating laboratory data, patient factors, and treatment choices.

The Effectiveness Gate comprises 13 metrics emphasizing the clinical value of decision-making, structured under a 70–20–10% weighting scheme for high-value diagnostic decisions, intermediate management tasks, and patient experience optimization, respectively. Approximately 85% of these metrics use graded scoring, requiring multidimensional case analysis, while a small proportion adopts binary evaluation for scenarios involving clear contraindications.

The metric development and weighting followed a two-stage expert consensus process.

Stage 1 – Metrics generation:

Seven senior clinicians and three medical informatics specialists proposed candidate metrics, which two LLM technical experts reviewed for feasibility and alignment with model‑evaluation objectives. Thirty metrics were finalized for Delphi weighting.

Stage 2 – Three-round Delphi weighting:

Seven senior clinicians independently rated each metric’s clinical risk weight on a 1–5 scoring scale. Ratings were summarized as medians. Metrics were re‑rated when the interquartile range exceeded 1 or panel agreement fell below 80%. After three rounds, all 30 items reached consensus.

To convert Delphi ratings into final weights (score-to-weight design), we mapped the weighted median and IQR to tiered weights. Safety gate: weighted median in [4.5, 5.0] with IQR ≤ 1 → weight 5 (life‑threatening/immediate fatality); [4.0, 4.5) with IQR ≤ 1 → weight 4 (life‑threatening/severe disability risk); [3.5, 4.0) with IQR ≤ 1 → weight 3 (severe disability risk/delayed treatment); [3.0, 3.5) with IQR ≤ 1 → weight 1 (moderate risk/reversible harm). Effectiveness gate: weighted median in [4.0, 5.0] with IQR ≤ 1 → weight 3 (high value/decision‑influencing); [3.5, 4.0) with IQR ≤ 1 → weight 2 (moderate value/management‑affecting); [3.0, 3.5) with IQR ≤ 1 → weight 1 (low value/experience‑oriented). Any metric with IQR > 1 triggered arbitration and scoped re‑evaluation; if IQR remained > 1, the metric was labeled “conditionally included/context‑specific” with constrained use scenarios (Supplementary Table 1).

### Model auto-scoring evaluation

For the automated evaluation of model responses to assessment points, we adopted the “LLM-as-Judge” paradigm^33,34^, utilizing a commercial large language model, Deepseek-R1, to construct the automated scoring engine. This evaluation framework comprises four core components: prompt design, question input, model response, and reference answers. All models were evaluated using temperature = 1.0, which provides a balance between determinism and diversity in output generation. All models were evaluated under standardized zero-context prompts, meaning that no external retrieval, dialog history, or contextual augmentation was provided. Each model received identical question stems and answer choices to ensure fairness and reproducibility.”

In view of our study’s primary focus on Chinese and the goal of improving model adherence to Chinese instructions, the dataset was not translated into English. Moreover, most mainstream Chinese LLMs are adapted from English baseline models and can process bilingual (Chinese–English) inputs without translation. To validate that translation does not materially affect the evaluation, we randomly sampled 210 cases for translation and assessed ranking consistency across two judge models (DeepSeek-R1 and GPT-4.1). We observed perfect rank-order agreement (Spearman ρ = 1.0) and a small mean absolute score difference with 95% CI of [0.2257, 0.2357] (Supplementary Table 10). Considering DeepSeek’s substantially lower inference cost and higher throughput, as well as the model’s stable availability that supports third-party replication of our evaluation pipeline and results, we selected DeepSeek-R1 as the primary scoring model.

The prompt design explicitly specifies the scoring rules for the 30 assessment metrics, including the scoring type for each item (binary or graded scoring), judgment criteria, and weight allocation. For binary classification items, the prompts define clear dichotomous criteria delineating the boundaries between “safe/compliant” and “unsafe/non-compliant” responses. For scored rating items, the prompts enumerate specific scoring dimensions such as relevance, completeness, and accuracy, along with the respective point allocations for each dimension. The question input consists of 2,069 clinical scenario questions to be evaluated, while the model response is the answer generated by the LLM under assessment. Reference answers and scoring guidelines are derived from standards developed and revised by 32 clinical specialists (DENT, IMG, and THBS each had 2 experts, ONC had 4 experts, and all other departments were represented by a single expert). For ONC (*n* = 4 experts, 70 cases), IMG (*n* = 2, 68 cases), THBS (*n* = 2, 65 cases), and DENT (*n* = 2, 56 cases), consensus was achieved through 2 iterative Delphi rounds. Notabley, at the specialty level, if the corresponding domain experts judged a given metric to be unsuitable or non-applicable for evaluation within that specialty (e.g., lacking clinical relevance or actionable risk signals in typical cases), the metric was not included in that specialty’s assessment set; consequently, not all specialty assessment sets contain all 30 metrics.

Agreement proportions improved from 89.0–92.3% (round 1) to 95.9–100% (round 2), meeting the convergence criterion of ≥ 85% agreement per department.This process aligns with modified Delphi (with elements of the Nominal Group Technique for structured feedback) for expert panel consensus. More information on participating clinicians can be found in Supplementary Tables 24, 25.

After revising, three medical informatics experts conducted a logic audit focused on whether each case and its standards complied with item-construction rules and ensured that all intended test points were embedded in the simulated cases; items with any issues were returned to the specialty physician for further revision until all cases achieved 100% approval.

To ensure reproducible and auditable evaluation, the scoring prompts are structured into eight explicit components: (1) role specification (defining the scorer’s expertise and neutrality), (2) task execution protocol (stepwise evaluation workflow), (3) input data description (what is provided and how it should be interpreted), (4) output format and conventions (including field names, types, and normalization rules), (5) output review protocol (self-checks and consistency validation before finalizing scores), (6) core evaluation rules (metric-wise criteria, thresholds, and scoring types), (7) core execution instructions (mandatory actions and prohibitions during scoring), and (8) final instruction (commit and emit the final structured score). The scoring inputs explicitly include the evaluation prompt itself, the clinical scenario questions (cases), the safety/effectiveness gate, and each assessed model’s response. The complete, executable prompts and templates will be provided on GitHub for public access and verification.

During the scoring process, the automated scoring LLM receives the question, the assessed model’s response, and the reference answers, then applies the scoring rules embedded in the prompts to assign scores. For binary classification items, a score of “1” (safe/compliant) or “0” (unsafe/non-compliant) is directly output based on whether the response meets the predefined standard. For scored rating items, the response is scored across each specified dimension according to the prompts, weighted accordingly, and summed to produce a total score. This total is then normalized to a 0–1 scale, where a higher score indicates greater alignment with clinical best practices.

To ensure scoring accuracy, prior to formal evaluation, the automated scoring engine was calibrated using a subset of samples. Calibration involved assessing agreement between automated and human scores using metrics such as the Kappa coefficient, which was predefined as a calibration criterion of κ ≥ 0.40 (moderate agreement)^35,36^ between the automated scorer and physicians; prompts and scoring rules were iteratively refined until this threshold was met.

### Binary score calculation criteria

A binary classification logic was applied, using absolute contraindications from clinical guidelines as the benchmark (e.g., drug contraindications for specific populations). A model response scored 1.0 if it fully adhered to the gold standard; any violation of contraindication principles resulted in a score of 0.0.

### Graded score calculation criteria

For evaluation scenarios requiring integration of clinical variables (e.g., dosage adjustments, differential diagnosis), a multi-rule weighted summation method was used. Each evaluation rule corresponded to a specific clinical criterion (e.g., lab values, symptom combinations) with a pre-assigned weight (1–5 points). The model score equaled the sum of the actual rule scores divided by the total possible rule scores, rounded to four decimal places and capped at 1.0. The scoring formula was:

1 $${Scor}{e}_{{Graded}}=\frac{{\sum }_{i=1}^{n}{r}_{i}}{{\sum }_{i=1}^{n}{s}_{i}}$$

where ${s}_{i}$ represents the score for the *i*th rule, ${r}_{i}$ the actual score achieved for that rule, and n the total number of rules. For example, in evaluating medication use for chronic kidney disease patients with rule weights of [5,4,3], if the model only correctly identifies the primary contraindication (scoring 5 points), the dynamic score is 5/(5 + 4 + 3) = 0.4167.

### Overall model score calculation

A weighted average method was used to aggregate scores across all test cases, with weight assignments directly linked to the clinical risk level of the associated metric (risk levels 1–5 corresponding to weight values of 1.0–5.0). The safety and effectiveness scores were obtained by aggregating scores within their respective gates. Multiple cases under the same metric were cumulatively weighted, ensuring that high-risk metrics (e.g., myocardial infarction emergency care) had 3–5 times the influence on the total score compared to low-risk metrics (e.g., health consultation). The total score formula was:

2 $${Scor}{e}_{{total}}=\frac{{\sum }_{i=1}^{n}{w}_{i}\cdot {Scor}{e}_{i}}{{\sum }_{i=1}^{n}{w}_{i}}$$

where ${Scor}{e}_{i}$ is the score of the *i*th test case, ${w}_{i}$ is the weight of the *i*th test case (reflecting the full weight of the corresponding metric), and n is the total number of test cases.

### Departmental score calculation

Scores were weighted and calculated across 26 departments using the same logic as the overall model score, but only incorporating cases relevant to each department (e.g., only pediatric cases were included in the pediatric department score). Additionally, stratified statistics were conducted by risk level (1–5) and gate type (safety/effectiveness). For instance, cardiovascular internal medicine safety gate scores for level 5 risk cases were calculated separately to identify performance gaps in high-risk specialty domains. The departmental score formula was:

3 $${Scor}{e}_{{dept}}=\frac{{\sum }_{j=1}^{k}{w}_{j}\cdot {Scor}{e}_{j}}{{\sum }_{j=1}^{k}{w}_{j}}$$

where ${Scor}{e}_{j}$ is the score of the *j*th test case in that department, ${w}_{j}$ is the weight of the *j*th test case (reflecting the full weight of the corresponding metric), and k is the total number of test cases in the department.

### data aggregation and mean calculation for model score comparison

Each model was evaluated independently three times on the same set of cases. The arithmetic mean of the three scores was taken as the case-level average score. The final model score was the weighted sum of these case-level averages, with weights consistent with those used in a single evaluation.

### Error estimation and visualization

We quantified scoring variability using 95% confidence intervals calculated via the bootstrap method (see “Code availability”). Error bars represent half the length of the confidence interval and are computed as the standard deviation of the bootstrap resamples divided by the square root of the sample size. Statistical significance testing employed two bootstrap-based approaches for p-value estimation: paired bootstrap was used for comparisons between different models on the same set of cases—calculating the mean of the original differences, then performing 10,000 bootstrap resamples with replacement on the difference array to generate the test statistic, followed by a two-tailed *p*-value calculation; independent bootstrap was applied to comparisons between different case cohorts—computing the original mean difference between groups, independently bootstrapping each group 10,000 times to obtain a distribution of differences, then calculating the two-tailed p-value. For the 15 pairwise comparisons among six models, multiple testing correction was performed using the Holm-Bonferroni method, which sequentially adjusts the original p-values sorted in ascending order.

### Evaluation of the impact of structured prompt engineering on model performance

To validate the impact of structured prompt engineering on model performance, we designed a standardized testing procedure that includes test set construction, model response generation, and optimization effect comparison. This method aims to rapidly assess the benefits of prompt optimization using a small but highly representative dataset that broadly covers medical scenarios.

We employed a balanced sampling strategy to construct a representative test subset from the original dataset of 2069 cases. The resulting test dataset consists of 60 representative cases (sourced from the 2069 original cases), covering all 30 assessment criteria and 26 specialty departments to ensure balanced sample distribution. To guide the model in generating structured, safe, and effective medical recommendations, we designed corresponding prompts (see Supplementary Note 2). During model interaction, these prompts served as system-level instructions, with each case from the test set provided as user input. The model responses generated under this framework were then evaluated to determine the practical effects of structured prompts on output quality.

Subsequently, the paired bootstrap test method described in the “Paired Bootstrap Testing by Case ID” section was employed for statistical analysis. This approach resamples the score differences for the same case before and after optimization to calculate the confidence interval and p-value of the performance improvement, thus determining the statistical significance of the optimization effect.

### Model repeatability evaluation method

We employed the Worst at k metric to assess model output stability and the risk of generating extremely low-quality results, following the process outlined below:

Test Set and Evaluation Rounds: From the 2069 original cases, 2 cases were randomly selected for each of the 30 assessment criteria, forming a test set of 60 cases. Each case was independently answered by the model 10 times, resulting in 10 distinct scores per case: {s_{1},s_{2},…,s_{10}}.

Worst at k Calculation: For a given k value (ranging from 1 to 10), k samples were randomly drawn without replacement from the 10 scores of each case, and the minimum score among them was recorded. The arithmetic mean of the minimum scores across the 60 cases was then computed, yielding the Worst@k score, defined as:

4 $${Worst}{\rm{@}}k=\frac{1}{M}{\sum }_{j=1}^{M}[{mi}{n}_{s\in {Sampl}{e}_{k}({R}_{j})}(s)]$$

where M is the total number of test cases (M = 60), ${R}_{j}$ represents the set of 10 scores for the *j*th case, and ${Sampl}{e}_{k}{(R}_{j})$ is the subset of k scores sampled from ${R}_{j}$ without replacement.

By calculating the Worst@k scores across different k values, we plotted performance degradation curves to compare model stability.

### Model scoring consistency evaluation method

Scoring consistency evaluation is a critical step in ensuring the reliability of the automated evaluation system, by quantifying the alignment between model scoring and human expert judgments. To comprehensively assess LLMs’ qualitative responses to diverse medical questions, we conducted a blinded human evaluation on 411 oncology-specific items by four board-certified specialists. The credibility of the scoring engine was validated through rule-based evaluation and outcome variability analysis using 4303 doctor-revised rules, enabling systematic verification of scoring logic and robustness across clinical scenarios:

Binary Scoring Consistency: We compared the model’s and doctors’ judgments for each binary rule using the following formula:

5 $${Agreemen}{t}_{{binary}}=\frac{{\sum }_{i=1}^{n}1[{Mode}{l}_{i}={Docto}{r}_{i}]}{n}$$

where n is the total number of evaluated rules, 1[⋅]is the metric function, and ${Mode}{l}_{i}$ and ${Docto}{r}_{i}$ denote the model’s and doctor’s judgments on the *i*th rule, respectively.

Graded Scoring Consistency: For cases involving multiple clinical variables, we compared the consistency of the model and doctors across multiple rules within each case:

6 $${Agreemen}{t}_{{dynamic}}=\frac{{\sum }_{j=1}^{m}{\sum }_{k=1}^{{c}_{j}}1[{Mode}{l}_{j,k}={Docto}{r}_{j,k}]}{{\sum }_{j=1}^{m}{c}_{j}}$$

where m is the total number of graded-type cases, ${c}_{j}$ is the number of rules in the *j*th case, and ${Mode}{l}_{j,k}$ and ${Docto}{r}_{j,k}$ are the model’s and doctor’s judgments on the *k*th rule of the *j*th case, respectively.

### Evaluation Metric: Macro F1 Score

We compared the model scorer’s predictions (“compliant”/“non-compliant”) with physician annotations to compute the Macro F1 score:

7 $$F{1}_{{positive}}=\frac{2\times {TP}}{2\times {TP}+{FP}+{FN}}$$

8 $$F{1}_{{negative}}=\frac{2\times {TN}}{2\times {TN}+{FN}+{FP}}$$

9 $${MacroF}1=\frac{1}{2}\times (F{1}_{{positive}}+F{1}_{{negative}})$$

where TP (True Positive): number of rules where both model and doctors judged as “compliant”; TN (True Negative): both judged as “non-compliant”; FP (False Positive): model judged “compliant” but doctors judged “non-compliant”; FN (False Negative): model judged “non-compliant” but doctors judged “compliant”.

The Macro F1 score between different doctors served as the baseline for human expert consistency, while random guessing (probability of compliance equal to the positive class frequency) set the lower bound (Macro F1 = 0.5). Inter-doctor consistency was calculated as:

10 $${Inter}-{DoctorF}1=\frac{1}{(\frac{D}{2})}\mathop{\sum }\limits_{i=1}^{D-1}\mathop{\sum }\limits_{j=i+1}^{D}{MacroF}1({Docto}{r}_{i},{Docto}{r}_{j})$$

where D is the total number of participating doctors.

### Ethics

All data sources we use to construct the Clinical Safety-Effectiveness Dual-Track Benchmark, CSEDB benchmark dataset, are publicly available and free to use without copyright infringement. All questions in the CSEDB dataset have been appropriately anonymized so that they do not contain sensitive private information about patients. We do not foresee any other possible negative societal impacts of this work.

## Supplementary information

Supplementary Information

## Data Availability

All the Supplementary Information and Appendix Tables used in the study are also available in the following repository: https://github.com/Medlinker-MG/CSEDB.

## Code availability

All code for reproducing our analysis is available in the following repository: https://github.com/Medlinker-MG/CSEDB.
